# Methylation of the Glucocorticoid Receptor (NR3C1) in Placenta Is Associated with Infant Cry Acoustics

**DOI:** 10.3389/fnbeh.2016.00100

**Published:** 2016-06-02

**Authors:** Stephen J. Sheinkopf, Giulia Righi, Carmen J. Marsit, Barry M. Lester

**Affiliations:** ^1^The Brown Center for the Study of Children at Risk, Women and Infants HospitalProvidence, RI, USA; ^2^Department of Psychiatry and Human Behavior, Warren Alpert Medical School of Brown UniversityProvidence, RI, USA; ^3^Department of Pediatrics, Warren Alpert Medical School of Brown UniversityProvidence, RI, USA; ^4^Section of Biostatistics and Epidemiology, Department of Pharmacology and Toxicology and of Community and Family Medicine, Geisel School of Medicine at DartmouthHanover, NH, USA

**Keywords:** infancy, human, placenta, epigenetic, neurodevelopment, cry, acoustics

## Abstract

Epigenetic mechanisms regulating expression of the glucocorticoid receptor gene (*NR3C1*) promoter may influence behavioral and biological aspects of stress response in human infants. Acoustic features of infant crying are an indicator of neurobehavioral and neurological status not yet investigated in relation to epigenetic mechanisms. We examined *NR3C1* methylation in placental tissue from a series of 120 healthy newborn infants in relation to a detailed set of acoustic features extracted from newborn infant cries. We identified significant associations of *NR3C1* methylation with energy variation in infants' cries as well as with the presence of very high fundamental frequency in cry utterances. The presence of high fundamental frequency in cry (above 1 kHz) has been linked to poor vocal tract control, poor regulation of stress response, and may be an indicator or poor neurobehavioral integrity. Thus, these results add to evidence linking epigenetic alteration of the *NR3C1* gene in the placenta to neurodevelopmental features in infants.

## Introduction

Early life experiences, beginning in the prenatal period, can result in persistent changes in an individual's responses to stress and challenge. Adverse experiences and environmental risks can impact and result in long-term alterations in stress response, including impacts on the hypothalamic-pituitary-adrenal axis (HPA) system (Welberg and Seckl, 2001). Epigenetic changes constitute one mechanism through which structural changes to DNA impact persistent gene expression patterns. There is a growing body of evidence that environmental factors in the pre- and perinatal period impact gene expression through epigenetic mechanisms (Liu et al., 1997; Francis et al., 1999; Weaver et al., 2004).

Epigenetic regulation refers to structural alterations of chromatin that impact gene expression without changing the underlying DNA molecular sequence. One commonly studied epigenetic mechanism is DNA methylation, which represents the presence of a methyl group on a cytosine followed by a guanine, known as a CpG site, which often exists in the promoter region of a gene. DNA methylation is one example of an epigenetic mechanism that is linked to gene expression (Bird et al., 1981; Bird, 1984).

The *NR3C1* gene encodes for the glucocorticoid receptor (GR) that is expressed in the brain and numerous peripheral tissues and is involved in binding cortisol and regulating cortisol response and levels. In animal models, the expression of the GR gene has been found to be increased in rodent offspring whose mothers spent more time engaged in licking, grooming and other aspects of maternal care (Liu et al., 1997; Francis et al., 1999). Further, rodent offspring whose mothers spent more time licking and grooming had lower methylation of the *Nr3c1* promoter region and greater GR gene expression in the hippocampus, and this was related to lower HPA stress responses in the adult offspring (Weaver et al., 2004). Recent research has examined the impact of prenatal stressors (e.g., maternal depression) on the methylation of *NR3C1* human newborns, and has demonstrated relations between specific epigenetic markers and measures of neurobehavior in the newborn period (Bromer et al., 2013; Lester et al., 2015) and stress response in infancy (Oberlander et al., 2014).

Thus, an emerging literature has linked epigenetic modifications of the *NR3C1* gene and stress response and regulation in human infants as well as animal models. The current study sought to examine the potential link between *NR3C1* methylation in placental tissue and additional neurobehavioral measures related to infant stress and autonomic response. Specifically, we examined the relation between infant cry acoustics and *NR3C1* methylation in a group of healthy full term newborn infants. In this model, the placenta, as a functional organ, is involved in fetal programming influencing the development of stress response systems that can be measured by postnatal neurobehavioral indicators (Ponder et al., 2011; Marsit et al., 2012a; Bromer et al., 2013; Conradt et al., 2015).

Infant cry serves a critical signaling function in human infants, and can be thought of as analogous to distress vocalizations in other mammalian species (Newman, 2007). Cries reflect distress and discomfort, and signal to caregivers in a way that supports caregiving and that is part of the emerging system of infant-parent communication (Lester, 1984). In addition to the developmental function of infant cry, individual differences in the quality and structure of infant cry is an indicator of an infant's health and neurological status. Numerous studies have found differences in acoustic elements of cries that are associated with specific medical conditions and risk factors (LaGasse et al., 2005). Relevant to the current study, research has also linked qualities of infant cries to other measures of stress response and regulation. Zeskind et al. (1996a) reported that infants with atypical response to pain stimuli had a longer latency to cry and produced cries with shorter utterances. These differences in cry production were related to diminished autonomic regulation of periodicities in cardiac rhythms (lower power in heart rate spectral analysis). Consistent with this finding, Stewart and colleagues reported an association between prosodic aspects of infant cry and autonomic state during a stressful experience (Stewart et al., 2013).

In this paper, we report an examination of infant cry utilizing a recently developed automated acoustic cry analysis (Reggiannini et al., 2013). Cries were elicited from infants in an awake state following a newborn exam, and acoustic features of these cries were extracted from digital audio recordings. The resulting acoustic analysis was examined in relation to *NR3C1* methylation analyzed from placenta samples collected at birth. This study builds on prior work that has utilized placental tissue for epigenetic analysis in relation to the prenatal, intrauterine environment (Novakovic et al., 2011; Xiao et al., 2016), and neurobehavioral features in the fetus and neonate (Marsit et al., 2012a; Bromer et al., 2013; Conradt et al., 2015; Monk et al., 2016). Our hypothesis was that greater methylation of CpG sites in the *NR3C1* gene would be related to acoustic features of infants' cries known to be reflective of greater neurobehavioral distress and neurological status/integrity.

## Methods

### Participants

Study subjects were drawn from the Rhode Island Child Health Study (Marsit et al., 2012b), which enrolls mother and infant pairs following delivery at the Women and Infants Hospital of Rhode Island (Providence, RI, USA). This study was carried out in accordance with the recommendations of institutional ethics boards at Women and Infants Hospital of Rhode Island and Dartmouth College. All mothers provided written informed consent.

Full term infants (≥37 weeks) were enrolled and included infants born small for gestational age (SGA, lowest 10th percentile), or large for gestational age (LGA, highest 10th percentile), based on birth weight and gestational age and calculated from the Fenton growth chart (Fenton, 2003). Infants appropriately sized for gestational age matched on sex, gestational age (±3 days), and maternal age (±3 years) were also enrolled. Exclusion criteria were non-singleton birth, maternal age < 18 or >40 years, presence of a life-threatening medical complication for the mother, and congenital or chromosomal abnormality of the infant. A structured chart review was used to collect information from the maternal and newborn inpatient medical record from delivery. Prior to hospital discharge, mothers completed a structured interview during which information regarding demographic characteristics and prenatal exposures was collected. For this analysis, the 120 participants who had available placental *NR3C1* methylation information and available cry acoustic information were examined. No participants from the analytic sample were exposed to synthetic glucocorticoids (e.g., prednisone, betamethasone, dexamethasone) during the prenatal period.

### Placenta sample collection, DNA extraction, and bisulfite pyrosequencing

Within 2 h of delivery, 12 fragments of placenta tissue were excised from each subject. Three tissue samples were taken from each of four placental quadrants (totaling approximately 1 g of tissue). Samples were full thickness sections of placental parenchyma 2 cm from the umbilical cord insertion site and were free of maternal decidua. Tissue samples were immediately placed in RNAlater solution (Life Technologies, Grand Island, NY) and stored at 4°C. At least 72 h later, placenta samples were removed from RNAlater, blotted dry and snap-frozen in liquid nitrogen. Samples from all regions were homogenized into a single sample using a mortar and pestle. The samples are stored at –80°C until needed for examination. DNA was extracted from homogenized placenta samples using the QIAmp DNA Mini Kit (Qiagen, Inc., Valencia, CA) following manufacturer's protocols and purified DNA was quantified using a ND-1000 spectrophotometer (Nanodrop, Wilmington, DE).

Genomic DNA was subjected to bisulfite modification using the EZ DNA methylation Kit (Zymo Research, Irvine, CA). Pyrosequencing of the *NR3C1* exon 1F region was performed on PCR product amplified from bisulfite modified DNA as described previously (Filiberto et al., 2011) The primers for amplification were Forward: 5′-TTT TTT TTT TGA AGT TTT TTT A-3′ and Reverse: 5′-Biotin-CCC CCA ACT CCC CAA AAA-3′. Two primers were used to sequence the amplification product in the 1F region, sequence 1: GAG TGG GTT TGG AGT-3′ and sequence 2: 5′-AGA AAA GAA TTG GAG AAA TT-3′. Percent DNA methylation at each CpG site was quantified using the Pyro Q-CpG software, version 1.0.11 (Qiagen). Bisulfite conversion controls were included on each sequencing read. In order for the sample's DNA methylation extent to be called, the bisulfite conversion rate must be >93%, and for all samples examined the conversion rate was >95%. All samples were sequenced in triplicates from the same bisulfite converted DNA template, and if the repeats differed by >10% the sample was repeated. These methods for quantifying DNA methylation from placental tissue have been used previously by our research group (Marsit et al., 2012b; Conradt et al., 2015).

It is noted that bisulfite based techniques cannot distinguish between DNA methylation and other similar modifications (e.g., hydroxymethylation). However, the occurrence of these other forms of modification is rare in fully differentiated tissues, and have been found in regions of the placenta genome other than those examined in this study (Green et al., 2016).

### Cry recording and acoustic analysis

Infant cries were recorded following administration of the NICU Network Neurobehavioral Scales (NNNS) (Lester et al., 2004). The NNNS is a standardized exam that assesses infants' neurobehavioral status. The NNNS was administered during the post-partum hospital stay. Exams were scheduled 30–60 min after a feeding and when the infant had been sleeping for 30 min to assure similar baseline states across infants. Immediately following the NNNS, infants were in an awake state allowing for standard elicitation of cry. Cries were elicited by a mildly painful poke to the bottom of the foot by the examiner while the infant lay supine in a bassinette. A maximum of two elicitations were conducted; a second stimulus was administered if the first did not result in arousing the infant into a full, crying state as defined by standard criteria (i.e., intense, loud, rhythmic, and sustained vocalizations that were differentiated from brief cries and fusses characteristic of lower states of arousal). Recordings were sampled at 48 kHz with an Olympus direct PCM recorder and saved as standard uncompressed audio files for later analysis.

These pain-elicited cries were analyzed using an algorithm that extracts acoustic parameters from standard digital audio files (Reggiannini et al., 2013). The system uses a 2-phase cepstral based acoustic analysis. A detailed description of the analysis method, including validation studies, has been previously reported (Reggiannini et al., 2013). The analysis is described briefly here. First, the system extracts acoustic information with a 12.5 ms frame advance, and then organizes and summarizes this information in cry utterances. Cry utterances were defined as a cry during the expiratory phase of respiration lasting at least 500 ms. Measures of interest include the standard deviation of the energy present in the signal, cry amplitude, the length of the utterance, the basic or fundamental frequency of the utterance (*F*_0_, which is perceived as “pitch”) and its related formants (harmonic content of a sound influenced by the mouth shape and vocal cord length), the presence of a fundamental frequency greater than 1 kHz (termed, “hyperpitch”), and information regarding the percentage of cry utterances with a clear harmonic structure (“voicing”) and the presence of turbulent noise (“frication”).

Each of the cry acoustic features yields a range of variables describing these characteristics across each utterance resulting in a large number of cry variables. In order to reduce the number of variables for analysis, a principal component analysis (PCA) with Promax oblique rotation was conducted on the first 3 cry utterances that each infant produced after the administration of a standardized painful stimulus. The resulting factor structure of the cries is described in the Results Section, below.

### Data analysis and hypothesis testing

The relationship between mean DNA methylation and acoustic properties of the cry signal was examining using Spearman rank correlations. Correlations were computed between mean DNA methylation in the *NR3C1* gene and PCA-derived cry factors. We tested the relation between the cry factors and the mean methylation across the 13 CpG sites in *NR3C1*. We followed these analyses with planned tests of the first 4 of the 13 *NR3C1* CpG sites in the 1F region (representing the following genomic coordinates: GRCh37/hg19 Chr 14: 142783501, 142783503, 142783513, 142783519), a region that has been shown to relate to HPA axis and stress reactivity in studies of rodents (Weaver et al., 2004) and humans (Oberlander et al., 2008). Multiple testing was accounted for by using the Benjamini and Hochberg False Discovery Rate method (FDR *q* < 0.10) (Benjamini and Hochberg, 1995).

In order to determine whether the associations between DNA methylation and the acoustic properties of the cry signal are independent of demographic factors, generalized linear models were computed. These models were constructed by assigning each of the PCA-derived cry factors as outcomes, and utilizing DNA methylation and potentially relevant demographics factors, including infants' sex, birth weight percentile, maternal age, tobacco use during pregnancy, and maternal education (used as a proxy for socioeconomic status), as predictors. Separate models were computed in order to verify the stability of the significant relationships identified by Spearman correlations between methylation ratios and PCA-derived cry factors. All analyses were carried out in SPSS v.22 (Armonk, NY).

## Results

### Demographics

The demographic characteristics of the 120 infants included in the study are shown in Table 1. Infants were roughly equally distributed among genders. Infants' average birth weight fell in the 64th percentile with a standard deviation of 30 percentile points. Average maternal age was 30 years, with a standard deviation of 5 years. The majority of infants were white (69%). The majority of mothers reported at least some college experience (72%). Only 5% of mothers reported tobacco use during pregnancy.

**Table 1:** **Demographic and descriptive statistics of study variables**.

**Variables**		**N**	**Mean (SD) or %**
Birth weight percentile		115	64 (32.2)
Maternal age		117	30.15 (5.1)
Infant gender	Male	61	51%
	Female	57	48%
Maternal ethnicity	White, Non-Hispanic	83	69%
	Black	10	8%
	Hispanic	2	2%
	Other	19	16%
	Unknown	6	5%
Maternal education	<11th grade	8	7%
	HS/GED	21	18%
	Some college	22	18%
	College graduate	44	37%
	Any post college	21	18%
	Unknown	4	3%
Maternal tobacco use	Yes	6	5%
	No	107	89%
	Unknown	7	6%

### PCA results

Results of the PCA analysis are shown in Table 2. In utterance 1, PCA extracted five components with eigenvalues greater than 2. These components accounted for a total of 69.8% of the variance in the cry signal. The first PCA-derived component, which explained 21.9% of the variance, was primarily characterized by a combination of variables representing the standard deviation of energy contained in the cry signal across all frequency bands. The second PCA-derived component, which explained 17.6 of the variance, was primarily characterized by a combination of variables related to the presence of hyperpitch (i.e., *F*_0_ > 1kHz). The third PCA-derived component, which explained 12.1% of the variance, was primarily characterized by a combination of variables related to the formants present in the signal. The fourth PCA-derived component, which explained 9.4% of the variance, was primarily characterized by a combination of variables related to *F*_0_ (pitch). The fifth PCA-derived component, which explained 8.8% of the variance, was primarily characterized by a combination of variables that quantified the morphology of the signal and contained information about voicing and frication.

**Table 2:** **Factor structure derived from Principal Component Analysis (PCA) for acoustic measures extracted from the first three cry utterances**.

**(A) Utterance 1**					
**Variables**	**1**	**2**	**3**	**4**	**5**
Total energy SD	0.913				
Energy band 1 SD	0.911				
Energy band 2 SD	0.69				
Energy band 3 SD	0.666				
Energy band 4 SD	0.848				
Energy band 5 SD	0.838				
Energy band 6 SD	0.832				
Average pitch				0.79	
Max pitch				0.758	
Min pitch				0.417	
Pitch SD				0.488	0.563
Average hyperpitch		0.894			
Max hyperpitch		0.907			
Min hyperpitch		0.853			
Hyperpitch SD		0.545			
Voiced amplitude average	0.602				
High-Voiced amplitude average		0.915			
Formant 1 average			0.819		
Formant 1 max			0.698		
Formant 1 min			0.62		
Formant 1 SD			0.454		−0.413
Formant 2 average			0.791		
Formant 2 max		0.611	0.764		
% Fricative frames		0.564			0.545
% Long fricative segments					0.474
% Voiced frames	0.482				−0.489
**PCA RESULTS**					
Eigenvalue	6.12	4.94	3.39	2.62	2.47
Variance explained	21.9%	17.6%	12.1%	9.4%	8.8%
**(B) Utterance 2**					
**Variables**	**1**	**2**	**3**	**4**	**5**
Total energy SD	0.866				
Energy band 1 SD	0.893				
Energy band 2 SD	0.579				
Energy band 3 SD	0.837				
Energy band 4 SD	0.865				
Energy band 5 SD	0.801				
Energy band 6 SD	0.74				
Average pitch				0.844	
Max pitch				0.825	
Min pitch				0.582	
Pitch SD				0.526	0.523
Average hyperpitch		0.864			
Max hyperpitch		0.872			
Min hyperpitch		0.832			
Hyperpitch SD		0.639			
Voiced amplitude average	0.838				
High–Voiced amplitude average		0.850			
Formant 1 average			0.79		
Formant 1 max			0.684		
Formant 1 min			0.635		
Formant 1 SD	–	–	–	–	–
Formant 2 average			0.781		
Formant 2 max		0.402	0.700		
% Fricative frames	–0.463	0.438			0.629
% Long fricative segments	–0.412				0.636
% Voiced frames	0.693				–0.408
**PCA RESULTS**					
Eigenvalue	6.72	5.21	3.39	2.76	2.01
Variance explained	23.9%	18.6%	12.1%	9.9%	7.2%
**(C) UTTERANCE 3**					
**Variables**	**1**	**2**	**3**	**4**	
Total energy SD	0.934				
Energy band 1 SD	0.938				
Energy band 2 SD	0.459	–0.434		0.489	
Energy band 3 SD	0.657	0.500			
Energy band 4 SD	0.901				
Energy band 5 SD	0.867				
Energy band 6 SD	0.81				
Average pitch	0.502			–0.62	
Max pitch	0.583	0.407		–0.409	
Min pitch				0.626	
Pitch SD	–	–	–	–	
Average hyperpitch		0.885			
Max hyperpitch		0.888			
Min hyperpitch		0.879			
Hyperpitch SD	0.523				
Voiced amplitude average	0.683	–0.474			
High-Voiced amplitude average		0.853			
Formant 1 average			0.879		
Formant 1 max			0.806		
Formant 1 min			0.81		
Formant 1 SD				0.421	
Formant 2 average	–	–	–	–	
Formant 2 max		0.611		0.509	
% Fricative frames		0.564			
% Long fricative segments				0.43	
% Voiced frames	0.563				
**PCA RESULTS**					
Eigenvalue	7.14	5.1	2.82	2.39	
Variance explained	25.2%	18.2%	10.2%	8.6%	

*PCA results are reported separately for each utterance*.

The analysis of the second cry utterance revealed a similar component structure, with a total of five components with eigenvalues >2, which explained a total of 71.7% of the variance in the cry signal. Similar to the first utterance, the first PCA-derived component, which explained 23.9% of the variance, was primarily related to the standard deviation in energy across frequency bands; the second PCA-derived component, which explained 18.6% of the variance, was primarily related to hyperpitch; the third PCA-derived component, which explained 12.1% of the variance, was primarily related to formants; the fourth PCA-derived component, which explained 9.9% of the variance was primarily related to *F*_0_; the fifth PCA-derived component, which explained 7.2% of the variance, was primarily related to voicing and frication.

In utterance 3, PCA extracted four components with eigenvalues greater than 2. These components accounted for 62.16% of the variance in the cry signal. The first PCA-derived component, which accounted for 23.9% of the variance, was primarily derived from a combination of energy standard deviation across frequency bands; the second PCA-derived components, which accounted for 18.6% of the variance, was derived primarily from hyperpitch variables; the third PCA-derived component, which explained 10.2% of the variance was derived primarily from formants variables; the fourth PCA-derived component, which explained 8.56% of the variance, was derived primarily from *F*_0_ variables.

### Mean NR3C1 methylation and cry results

Descriptive data (mean, SD, range) for *NR3C1* methylation are presented in Table 3. In order to examine associations between PCA-derived cry factors and mean *NR3C1* methylation ratios, Spearman rank correlations were computed, the results of which are presented in Table 4. For utterance 1, mean methylation of the *NR3C1* gene was negatively associated with the variation in acoustic energy of the cry signal (ρ = −0.292, *p* = 0.002, *q* = 0.007). Mean methylation was not associated with the other cry factors for utterance one. For utterance 2, mean methylation of the *NR3C1* gene was negatively associated with the *F*_0_ factor (ρ = −0.276, *p* = 0.003, *q* = 0.021). The correlation between mean *NR3C1* methylation and cry energy variation in utterance 2 was significant at the FDR corrected threshold (ρ = −0.206, *p* = 0.028, *q* = 0.029). In utterance 3, mean methylation of the *NR3C1* gene was negatively associated with energy variation in the cry signal (ρ = −0.300, *p* = 0.002, *q* = 0.014). Mean methylation was also negatively associated with hyperpitch, uncorrected, but not after FDR correction (ρ = −0.202, *p* = 0.037, *q* = 0.036).

**Table 3:** **NR3C1 mean methylation, standard deviation, and range for CpG sites 1 to 13**.

**CpG Position**	**Mean**	**SD**	**Minimum**	**Maximum**
Position 1	0.86	1.31	0.00	8.2
Position 2	0.86	1.28	0.00	8.81
Position 3	1.04	1.14	0.00	4.23
Position 4	0.75	1.2	0.00	6.22
Position 5	0.94	0.9	0.00	3.02
Position 6	0.84	1.31	0.00	6.7
Position 7	3.28	0.77	2.14	7.26
Position 8	1.34	0.83	0.00	7.03
Position 9	2.33	1.8	0.00	18.2
Position 10	2.24	1.11	0.00	6.95
Position 11	1.56	1.1	0.00	4.58
Position 12	2.26	0.73	0.00	5.29
Position 13	1.64	0.85	0.00	4.49

**Table 4:** **Correlations (Spearman rho) between cry factors and NR3C1 mean methylation and methylation at CpG sites 1 to 4**.

	**Energy Variation**		**Hyperpitch**		**Formants**		**Pitch**		**Voicing**	
**CpG Position**	**rho**	***p***	**rho**	***p***	**rho**	***p***	**rho**	***p***	**rho**	***p***
**(A) CRY UTTERANCE 1**										
Mean^#^	−**0.292**	0.002*	0.094	0.320	0.060	0.520	−0.150	0.110	−0.002	0.990
Position 1	−**0.485**	<0.001*	**0.306**	<0.001*	−**0.261**	0.005*	−0.011	0.910	**0.195**	0.037*
Position 2	−**0.469**	<0.001*	**0.314**	<0.001*	−0.097	0.304	−0.091	0.330	0.177	0.059
Position 3	−**0.342**	<0.001*	**0.308**	<0.001*	−0.115	0.222	−**0.208**	0.027*	**0.227**	0.015*
Position 4	−**0.393**	<0.001*	**0.223**	0.017*	−0.106	0.261	−0.015	0.877	0.062	0.515
(**B) CRY UTTERANCE 2**										
Mean^#^	−**0.206**	0.028*	0.051	0.590	−0.087	0.356	−**0.276**	0.003*	0.016	0.865
Position 1	−**0.358**	<0.001*	**0.360**	<0.001*	0.007	0.944	−0.179	0.056	0.167	0.075
Position 2	−**0.248**	0.008*	**0.269**	0.004*	−0.042	0.659	−0.162	0.085	**0.191**	0.042*
Position 3	−**0.207**	0.027*	0.120	0.202	−0.008	0.936	−0.154	0.101	**0.197**	0.036*
Position 4	−**0.190**	0.043*	0.129	0.172	0.128	0.175	−0.155	0.100	0.075	0.429
**(C) CRY UTTERANCE 3**										
Mean^#^	−**0.300**	0.002*	−0.202	0.037	−0.115	0.237	−0.079	0.417		
Position 1	−**0.418**	<0.001*	−**0.274**	0.004*	−0.102	0.296	**0.235**	0.015*		
Position 2	−**0.362**	<0.001*	−0.163	0.093	−0.808	0.412	**0.331**	0.001*		
Position 3	−**0.334**	<0.001*	−**0.236**	0.014*	−0.077	0.429	0.117	0.232		
Position 4	−**0.302**	0.002*	−**0.215**	0.026*	−0.019	0.847	0.074	0.449		

# *Mean methylation across all 13 NR3C1 CpG sites. Values in bold and with an asterisk are significant at q < 0.10, FDR threshold (14 tests for the mean methylation findings and 56 tests for CpG sites 1 to 4 in relation to cry utterances 1, 2, and 3)*.

In order to test whether the relationships detected between methylation ratios and cry factors were confounded by demographic variables, generalized linear models were computed to follow up on the significant correlations between methylation ratios and cry parameters. For each of the models, cry factors were included as outcomes, whereas methylation ratio, infant's gender, birth weight percentile, maternal age, tobacco use during pregnancy (present or absent), and maternal education (high school or less vs. any college and above) were included as predictors.

In utterance 1, the association between energy variation in the cry signal and mean methylation ratio remained significant after controlling for covariates (β = −3.952, *p* < 0.001). In utterance 2, the relationship between energy variation and mean methylation ratio was significant in the fully adjusted model (β = −2.772, *p* = 0.003), as was the relationship between the cry *F*_0_ factor and mean methylation ratio (β = −3.122, *p* = 0.001). In utterance 3, the relationship between energy variation and mean methylation ratio was significant in the fully adjusted model (β = −3.122, *p* = 0.001). However, the relationship between the cry hyperpitch factor and mean methylation ratio did not maintain statistical significance after controlling for covariates (β = −1.376, *p* = 0.179).

### Methylation at NR3C1 sites 1 to 4 and cry results

Following prior research that has found relationships between stress response and methylation in the first 4 CpG sites of *NR3C1* in rodent and human offspring (Weaver et al., 2004; Oberlander et al., 2008; Lester et al., 2015), Spearman rank correlations were computed to test the relations between these specific CpG sites and the cry factors from utterances 1 to 3. This resulted in 56 tests and multiple testing was controlled by FDR correction compared to a q^*^ set at 0.10. These results are also reported in Table 4. As can be seen, there were consistent correlations between methylation at these CpG sites and variation in cry energy in all three utterances (greater methylation corresponded to lower variation in cry energy). Moreover, although hyperpitch in cries was not correlated with the overall mean methylation, there was a pattern of positive association with hyperpitch and methylation at CpG sites 1, 2, 3, and 4 in the first cry utterance. This association was also seen for cry utterance 2 for CpG sites 1 and 2. Thus, the presence of hyperpitch in the first two utterances of infants' cries, an indicator poor vocal tract control and poor regulation of stress response (LaGasse et al., 2005), was associated with increased methylation of CpG sites shown previously to be related to stress response in humans and in animal models. In contrast to utterances 1 and 2, there were significant but lower in magnitude correlations between hyperpitch in utterance 3 and methylation for CpG sites 1, 3, and 4 in utterance 3, and in the reverse direction than was found for the first two cry utterances. Additional, though less consistent relations were found between methylation at CpG sites 1 to 4 and cry formants, pitch (*F*_0_), and voicing. These results can be seen in Table 4.

## Discussion

There is a long history of research linking differences in infant cry acoustics to medical risks and developmental conditions (LaGasse et al., 2005). To our knowledge this is the first study relating infant cry acoustics to epigenetic alterations. Research and theory has also linked individual differences in cry production to aspects of arousal, stress response and autonomic regulation in early infancy. Classic research on infant cry linked differences in cry acoustics to neural damage or specific genetic conditions such as cri du chat (e.g., Karelitz and Fisichelli, 1962; Vuorenkoski et al., 1966). Other research has linked differences in cry acoustics to pre- and perinatal risk factors including malnutrition (Lester, 1976), preterm birth (Michelsson et al., 1983), prenatal drug and alcohol exposures (Lester et al., 1991; Corwin et al., 1992; Zeskind et al., 1996b), and developmental disorders such as autism (Sheinkopf et al., 2012; Esposito et al., 2013). These pre- and perinatal risks are related to disruptions in arousal and regulation including disruptions in stress response that are influenced by the HPA system. Cry production, including differences in *F*_0_ (pitch), the energy or force of cry, and other acoustic features are also influenced by and thus an indicator of stress response in infants (Zeskind et al., 1996a; Newman, 2007; Stewart et al., 2013).

Prior research in animal models and in human studies has found that early environmental experiences impact arousal, regulation, and stress response, and that these impacts are in part mediated by epigenetic mechanisms. Differences in methylation of genes related to stress response systems have been found to correlate with individual differences in neurobehavioral responses and stress response in infants (Bromer et al., 2013; Oberlander et al., 2014). Infant cry is an additional neurobehavioral measure that may be related to variation in the expression of genes related to stress response. In the current paper we tested the hypothesis that variation in methylation ratios in the *NR3C1* gene that codes for GR would be related to variation in cry acoustics in a sample of full term infants assessed in the neonatal period.

Our findings revealed that higher mean levels of methylation in the *NR3C1* gene was associated with variation in cry energy (force of cry) and acoustic features related to the fundamental frequency (*F*_0_) of pain-elicited cries, especially acoustic features reflecting the presence of the extreme of *F*_0_ (hyperpitch). These associations between infant cry acoustics and *NR3C1* methylation were independent of the effects of demographic characteristics, birth weight, or prenatal exposure to tobacco (there were no exposures to illicit substances in this sample). Thus, the results of this paper add to the growing evidence linking epigenetic mechanisms to behavioral differences in human infants. While correlational in nature, findings such as those reported here are consistent with animal models that have been able to explore the potential mechanisms linking epigenetic markers in stress response related genes to both gene expression and behavioral changes (Weaver et al., 2004). *NR3C1* methylation was measured in placental tissue, and in this study the placenta is seen as a functional indicator of processes involved in fetal programming (Monk et al., 2016). Prenatal experiences impact the intrauterine environment by altering placental functioning via epigenetic changes to *NR3C1* (and other genetic sites). The potential effect of these changes on later neonatal neurobehavior, including cry production, can then be interpreted in the context of prenatal origins of health and disease (Gillman, 2005).

A central finding of this study was a consistent pattern of association between the presence of hyperpitch in cry utterances 1 and 2, and higher methylation in the first 4 CpG positions of the *NR3C1* gene. Hyperpitch, which has also been termed “hyperphonation” in classic work on infant crying (Truby and Lind, 1965), describes portions of a cry episode where the base frequency of the cry (*F*_0_) is >1 kHz. Hyperpitch, together with alterations in the force or energy of cry, are indications of poor regulation of stress response (LaGasse et al., 2005). In prior research, the presence of elevations in *F*_0_ > 1 kHz has been found in relation to serious medical conditions, brain injury, or perinatal risk factors (Wasz-Hockert et al., 1985; Furlow, 1997). The infants in the current study were healthy newborns without such obvious medical conditions or risks. What then might the relation between NR3C1 methylation and hyperpitch indicate in healthy, low risk infants? The presence of hyperpitched portions of cry may be indicative of poor neural or neurobehavioral integrity without frank brain damage. Elevated *F*_0_ may be found in infants who were exposed to stressful events in early development, including the prenatal period (Douglas and Hill, 2013), and it can be hypothesized that these infants may later exhibit regulatory difficulties (Zeskind et al., 1996a; Stewart et al., 2013). Thus, the association between hyperpitch and methylation at CpG sites 1 to 4 of the *NR3C1* gene is consistent with models linking epigenetic regulation of glucocorticoid receptor gene expression and neurological integrity and/or neurobehavioral markers of stress response in the neonatal period. An alternative or additional possibility is that the differences in pain sensitivity could be impacting cry acoustics, and that epigenetic differences in *NR3C1* may influence the infants' responses to the pain stimulus used to elicit cries. This possibility is worthy of further inquiry, although it is noted that the pattern of acoustic features appear to be more complex than would be typically used to characterize cries solely on the basis of being the result of the infant experiencing pain.

These first four CpG sites comprise a canonical binding element for the NGF1-A transcription factor that is important for brain growth and development including differentiation and neuronal plasticity. Methylation of this region has been shown to reduce NGF1-A binding and activation of *NR3C1* transcription (McGowan et al., 2009) thereby providing a possible mechanistic explanation for understanding known relations between acoustic features such as hyperpitch and neural integrity. These findings are consistent with other research that has linked methylation in these early *NR3C1* CpG sites to poor stress response in humans and animal models (Weaver et al., 2004; Oberlander et al., 2008), and to neurobehavioral risks such as premature birth (Lester et al., 2015) and maternal anxiety and depression during pregnancy (Oberlander et al., 2014).

A unique aspect of this study was the manner in which cry acoustics were analyzed and the use of factor analysis to characterize the resulting acoustic measures extracted from the cry samples. Cries were elicited in a standardized manner, with the same eliciting stimulus always following the same newborn exam. The acoustic cry analysis was conducted using a recently developed automated cry analysis system that utilizes well-validated algorithms to extract acoustic features and organize these features for analysis (Reggiannini et al., 2013). Moreover, the use of principal component analysis was utilized to identify a factor structure for the cry variables across the first three utterances of cry bouts. This is a relatively novel method with respect to infant cry analysis. Stewart et al. (Stewart et al., 2013) used factor analysis (PCA) to extract summary measures of cry acoustics of 6-month-old infants in relation to autonomic measures during social challenge. Stewart and colleagues found that autonomic reactivity (changes in heart period and heart rate variability) was related to reduced modulation of acoustic features in infants' cries. The results presented here are broadly consistent with these findings, indicating that reduced variability in cry energy and elevations in fundamental frequency above 1 kHz was related to methylation at CpG sites known to play a role in regulating stress response systems in humans. Work by others has also linked individual differences in cry production to disruptions in infants' regulatory responses to stress (Zeskind et al., 1996a).

Limitations of this study are acknowledged. First, while the relationship between infant cry acoustics and *NR3C1* methylation is consistent with more mechanistic findings from animal models, the results are correlational. Complementary evidence could be garnered from cry and methylation studies of infants with known prenatal risk factors such as elevated exposure to factors related to maternal experiences of stress, anxiety, or stress during pregnancy. Additional evidence that would strengthen these findings would be outcome measures of stress response (e.g., measures of heart rate variability; cortisol reactivity, etc). Conradt and colleagues have recently reported that in infants of mothers with depressive symptoms, the level of *NR3C1* methylation was moderated by the degree to which mothers displayed sensitive maternal behaviors (Conradt et al., 2016), and that this pattern of exposure to maternal depression with more vs. less maternal sensitivity was also related to infant cortisol levels. The findings reported here are consistent with Conradt et al.'s findings, as well as with other research linking methylation of the *NR3C1* gene and stress response systems. Finally, and in a related manner, measures of infant outcomes would potentially strengthen the interpretation that individual differences in cry acoustics, especially variation in the force (energy) of cry and the presence of hyperpitch in cry utterances, is indicative of stress and regulatory response in infants, or indicative of poor neurological status, and thus potentially part of a downstream set of neurobehavioral features influenced by the expression in the brain of genes that impact stress response systems.

It has been suggested that epigenetic mechanisms that influence the HPA stress response system may lead to atypical neurobehavioral responses in neonates, and in turn may be related to poor developmental outcomes (Lester et al., 2015). The finding that methylation of the *NR3C1* gene is related to cry acoustics is consistent with this general assertion, and the specific link to aspects of cry acoustics known to be related to neurologic integrity is also consistent with hypotheses that link epigenetic mechanisms and later developmental outcomes. The addition of cry acoustics to the behavioral epigenetic literature has the potential to enrich this literature. Infant crying is seen as both a biological indicator of infant status and as a communicative signal that elicits and influences parenting responses, a framework that has been described as a biosocial model of infant cry (Lester, 1984). Thus, the findings reported in this study suggest two pathways that can link epigenetic modifications of the *NR3C1* gene and potential developmental outcomes. The first is a biological model whereby individual differences in cry acoustics are indicative of developmental risk. The second is a biosocial model whereby the biological drivers of differences in cry acoustics impact the developing infant-parent communication system and relationship, in turn influencing developmental outcomes. Future research that replicates these findings and that examines such alternate pathways to developmental outcomes would enhance our understanding of potential mechanisms linking early biological processes to later behavioral developmental outcomes in infancy.

## Author contributions

SS–wrote the first draft of this manuscript and no honorarium, grant, or other form of payment was given to him or anyone to produce this manuscript and also had overall responsibility for the cry acoustic analyses. GR–was responsible for processing of the acoustic analyses, provided analysis of the data and assisted in writing and preparing the initial draft of the manuscript. CM–designed the study, was responsible for DNA extraction and sequencing and participated in writing the manuscript. BL–designed the study, participated in analysis and interpretation of the data, as well as writing the manuscript. All authors were involved in the writing of this manuscript and each author has seen and approved the submission of this version of the manuscript and takes full responsibility for the manuscript as well as the decision to submit this paper for publication.

## Funding

This study was supported by the National Institute of Mental Health R01MH094609 (to CM). The content is solely the responsibility of the authors and does not necessarily represent the official views of the National Institute of Mental Health, National Institute on Drug Abuse, or the National Institutes of Health.

### Conflict of interest statement

The authors declare that the research was conducted in the absence of any commercial or financial relationships that could be construed as a potential conflict of interest. The authors disclose a pending patent application relevant to the cry analysis system used in this research (Serial number: 14/633,224; Title: An accurate analysis tool and method for the quantitative acoustic assessment of infant cry).
